# Identification of a novel InDel locus within the *ACSL5* gene and its association with body measurement traits in goats

**DOI:** 10.5194/aab-68-171-2025

**Published:** 2025-03-03

**Authors:** Ao Wang, Xianfeng Wu, Qian Xu, Benzhi Wang, Tianfang Xiao, Yuan Liu, Wenyang Li

**Affiliations:** 1 Fujian Provincial Key Laboratory of Animal Genetics and Breeding/Institute of Animal Husbandry and Veterinary, Fujian Academy of Agricultural Sciences, Fuzhou, Fujian 350013, China; 2 College of Animal Science, Fujian Agriculture and Forestry University, Fuzhou, Fujian 350002, China; a current address: Institute of Animal Husbandry and Veterinary, Fujian Academy of Agricultural Sciences, Fuzhou, Fujian 350013, China

## Abstract

The *ACSL5* (acyl-CoA synthetase 5) gene plays a crucial role in the biosynthesis of triglycerides, phospholipids, and cholesterol as well as the metabolism of fatty acids, and goats derive approximately 80 % of their energy from volatile fatty acids. However, there is a limited number of studies on the impact of InDel (insertion/deletion) mutations in the *ACSL5 *gene on goat traits. Therefore, This study investigated the spatiotemporal expression pattern of *ACSL5* in goats and the association between its polymorphism and growth traits, aiming to identify useful DNA markers and provide a basis for the application of marker-assisted selection (MAS) in goat breeding. The qPCR technique was employed in the expression profiles of the *ACSL5* gene in Fuqing (FQ) goats. The PCR technique was employed for type detection of the InDel locus of the *ACSL5* gene in 528 goats. We analyzed the genotype frequency, allele frequency, polymorphism information content (PIC), and Hardy–Weinberg equilibrium (HWE) of the InDel locus of the *ACSL5* gene in goats. A general linear model was used to analyze the relationship between the genotypes and body size traits of goats. qPCR analysis indicated that the *ACSL5* gene was expressed ubiquitously in the nine tested tissues of FQ goats. The expression level of *ACSL5* in fat tissue at birth was higher than in adult ( P<0.01 ) and weaning ( P<0.05 ) goats. An InDel polymorphism termed del41712 was detected within the fifth intron of the *ACSL5* gene. Genetic screening revealed only two genotypes, ID and II, present in the three studied goat breeds. Association analyses conducted on 528 goats linked this InDel polymorphism to body measurement traits, showing significant associations with chest depth (ChD) in FQ goats ( P<0.05 ) and with body height (BH), body length index (BLI), and chest circumference index (ChCI) in Nubian (NB) goats ( P<0.05 ). These findings suggest that InDel mutations in the goat *ACSL5* gene may serve as a valuable molecular genetic marker for breeding programs aimed at improving production traits.

## 1 Introduction

The performance and quality of goats can be influenced by numerous factors, such as breed, genetics, post-slaughter processing, sex, age, and nutritional levels. Additionally, interactions between these variables can further modify these attributes. Of the most impactful of these, breed plays a substantial role in body measurement traits and meat quality traits given the influence of polymorphisms, gene expression and regulation, and epigenetic modifications that govern growth and development (Kim et al., 2019). Through the natural environment and artificial selection, many excellent varieties have been selected and bred, and for some local varieties there is still a lot of room for improvement despite the fact that they already have better growth characteristics. This plays an important role in the conservation of biodiversity and the development of local economies (Zhao et al., 2024). However, traditional breeding improvement methods are long, slow, and subject to genetic evolutionary instability. In contrast, marker-assisted breeding is highly accurate and efficient. Through deeper research and analysis of genes in ruminants, genes related to specific traits can be identified, thereby promoting genetic improvement of target traits and greatly improving breeding efficiency. Single nucleotide polymorphism (SNP) variation, insertion/deletion (InDel), and copy number variation (CNV) can rapidly identify mutations associated with growth traits and have been widely used in goat and other livestock breeding (Wang et al., 2022). It was found that the InDel mutation sites of the *CFAP43* and *RORA* genes in Shanbei white cashmere goats were significantly correlated with body measurement traits such as body height and body length (Mi et al., 2022; Zhou et al., 2023). These traits are very important for the breeding and production performance of goats.

The Fuqing (FQ) goat breed, a prominent local breed from China's eastern coast, exhibits resilience to coarse feed, shows robust adaptability to environmental changes, and produces succulent meat (intramuscular fat – IMF – more than 3.0 %) (Liu et al., 2019), which is a major meat quality trait that influences aroma, tenderness, and juiciness (Huang et al., 2023). Our previous study revealed that the intramuscular fat content in FQ goats is considerably higher than in Nubian (NB) goats (Liu et al., 2024). The NB goat, introduced to China from the Nubia region of Africa, has a fast growth rate and is used for genetic improvement of local goat breeds in southern China (Tao et al., 2020). Previous work by the research group confirmed significant differences in growth phenotypes and meat quality between the two breeds, with NB goats having greater body weights and a lower IMF content than FQ goats. The significant differences in growth and meat quality traits between FQ goats and NB goats provide an ideal model for investigating goat production. In this study, we sequenced the transcriptome of skeletal muscle from both goat breeds and identified important candidate genes, including *ACSL5* (acyl-CoA synthetase 5), *FGF7* (fibroblast growth factor 7), and *ITGAD* (integrin subunit alpha D), which may influence body measurement traits in goats (Liu et al., 2024).

The ACSL family encompasses five genes: *ACSL1*, *ACSL3*, *ACSL4*, *ACSL5*, and *ACSL6* (Kornberg and Pricer, 1953). The protein encoded by the *ACSL5* gene is instrumental in catalyzing the transformation of fatty acids into acyl-CoA esters (Luo et al., 2023) and exhibits widespread expression across mammalian organs, including the liver, small intestine, fat tissue, spleen, uterus, lungs, and skeletal muscle (Pérez-Núñez et al., 2019). The human *ACSL5* gene was first charted in 1998 (Lopes-Marques et al., 2013) in the chromosome 10's q25.1–q25.2 region (Oikawa et al., 1998). It has been posited that the *ACSL5* gene is instrumental in activating dietary long-chain fatty acids and modulating fatty acids in the human jejunum (Meller et al., 2013). In humans, *ACSL5* exists in three isoforms: a long isoform (739 amino acids), a shorter isoform (683 amino acids), and a rare isoform (659 amino acids), the last of which, marked by a deletion of 72 base pairs corresponding to exon 20 (Yamashita et al., 2000), might be involved in the pathogenesis of aberrant cell growth and migration (Gassler et al., 2007; Matesanz et al., 2016). *ACSL5* is critical for the biosynthesis of triglycerides, phospholipids, and cholesterol as well as for fatty acid metabolism (Paul et al., 2014). Overexpression of *ACSL5* can enhance fatty acid oxidation and the generation of free radicals while dampening insulin signaling in human muscle tubules (Kwak et al., 2019), whereas *ACSL5* gene disruption can impair neutral lipid secretion by the liver (Bu and Mashek, 2010). Research has shown that ablation of *ACSL5* mice increases hepatic and serum *FGF21* levels, reduces adiposity, improves insulin sensitivity, increases energy expenditure, and delays triglyceride absorption (Bowman et al., 2016). Furthermore, the activity of *ACSL5* can also influence gut microbiome composition, thereby impacting lipid metabolism (Sheng et al., 2018).

Considering that goats derive approximately 80 % of their energy from volatile fatty acids (Li et al., 2021), the *ACSL5* gene plays a crucial role in fatty acid metabolism. However, few studies have investigated the relationship between the *ACSL5* gene and body measurement traits in goats. This study explains the identification of a novel InDel locus within the *ACSL5* gene and its association with body measurement traits in goats. The InDel locus is significantly correlated with the body measurement traits of Fuqing and Nubian goats and can serve as an important molecular marker for improving goat body measurement traits.

## 2 Materials and methods

The protocol (protocol no. 202207FJ002) of the Faculty of Animal Policy and Welfare Committee of the Fujian Academy of Agricultural Sciences (FAAS) for the use and care of animals in research was followed throughout all of the experimental procedures.

### 2.1 Samples and data collection

A total of 528 blood samples of healthy adult female goats was collected, including 123 FQ goats, 286 NB goats, and 119 Jianzhou Daer (JZ) goats. To detect variations in the *ACSL5* gene, the selected goats were approximately 2 years old and had been fed the same diet under the same environmental conditions. All the animals had their growth data collected, including their body weight (BW), body height (BH), body length (BL), chest circumference (ChC), chest width (ChW), chest depth (ChD), hucklebone width (HhW), and cannon circumference (CaC), thus providing a method for measuring these qualities (Gilbert et al., 1993). Based on the recommendations of our previous research, the following metrics were also computed: trunk index (TI), body length index (BLI), chest circumference index (ChCI), cannon circumference index (CaCI), chest width index (CWI), and hucklebone width index (HuWI) (Wu et al., 2014).

In addition, nine Fuqing goats (three each at birth, at weaning, and in adulthood) were fed and slaughtered on an FAAS goat farm. Nine tissue types, i.e., the heart, liver, spleen, lungs, kidneys, leg muscle, longissimus dorsi muscle, fat, and brain, were collected for qPCR. All the samples were frozen in liquid nitrogen and stored at -80 °C.

### 2.2 Extraction of genomic DNA and RNA

The blood DNA from all the individual goats was extracted using the conventional phenol-trichloromethane method and diluted to 50 ng µ L^−1^ after determining the concentration and purity of all DNA samples using a nucleic acid concentration tester (Nanodrop, USA). The samples were then stored at -80 °C in a refrigerator for spare parts. To establish genomic DNA pools, a total of 50 DNA samples was randomly selected from each breed. These DNA pools were employed to screen InDel variations in the *ACSL5* gene using the PCR and sequencing techniques.

The total RNA was extracted from all individual tissues using the TRIzol method, and the RNA concentration was quantified using a NanoDrop 1000 spectrophotometer (Thermo Fisher Scientific Inc., Wilmington, DE, USA). Reverse-transcription PCR (RT-PCR) was then employed to reverse-transcribe the extracted RNA into cDNA using the TransScript®Uni All-in-One First-Strand cDNA Synthesis SuperMix for qPCR (One-Step gDNA Removal) kit (Transgene Biotechnology Co., Ltd.). The reaction system consisted of 50 ng RNA, a 4 µ L 5 × TransScript^®^ Uni All-in-One SuperMix for qPCR, 1 µ L gDNA Remover, and 14 µ L of RNase-free water. The reaction was initiated by mixing the components and was incubated at 50 °C for 5 min. Following this, the reaction was incubated at 85 °C for 2 s. Then the samples were stored at -80 °C.

### 2.3 Primer synthesis

Based on the sequence data and variation details of the *ACSL5* gene (NC_030833.1) obtained from the NCBI (https://www.ncbi.nlm.nih.gov/, last access: 12 May 2024) and Ensembl (http://asia.ensembl.org/index.html, last access: 12 May 2024) databases, the primer pair for predicted mutation locus detection and analysis of the *ACSL5* gene expression quantity was designed using the NCBI online primer design software (https://www.ncbi.nlm.nih.gov/tools/primer-blast/index.cgi?LINK_LOC=Blast Home Primer, last access: 13 May 2024) and primer premier 5.0 (Table 1). The primers were synthesized by Biosune Biotechnology Co. (Fuzhou, China). The PCR product was sequenced by Shanghai Sangong Biotechnology Co. (Shanghai, China) using the Sanger sequencing method.

**Table 1:** Information on the primers.

Primer	Sequence of the primer (5^′^–3^′^)	Temperature (°C)	Product size (bp)	Identified site
P1	F:TCTTGCTGTGCCCTCACTTGA	62	339	ins40924
	R:AGGAGACCCTGGTTCAATTCCTG			
P2	F:TTGCTGTGCCCTCACTTGACT	61	373	ins40924
	R:CCACTCCAGTATTCTTAGGCTTCC			
P3	F:ATTCATCTTCCCTTAACCCAGCC	60	269	ins40672
	R:AAAGTCAAGTGAGGGCACAGC			
P4	F:TCTGTGCTGTGCTGTGCGTA	62	338	ins40924
	R:AGGAGACCCTGGTTCAATTCCTG			
P5	F:TCCAGGTAGCCTTACTCCAC	58	356	ins41658
	R:CAACAAATGCTTATTTGGTGGCT			
P6	F:TCCCAAAGTAGTTCCAGGTAGC	60	369	ins41658
	R:TCAACAAATGCTTATTTGGTGGCTC			
P7	F:ACACAGAGCCACCAAATAAGCATT	61	589	ins42222
	R:TTGGTGCATGCTGGCGAAC			
P8	F:CCACCAAATAAGCATTTGTTGACTA	58	575	ins42222
	R:CATGCTGGCGAACTATACTCAT			
P9	F:CTGGGTTGGAATTTGGGTTGTA	59	632	ins44213
	R:AGTGAAGCCATTCCAGTGATTG			
P10	F:GGGTCACTTGGGGTCTTCTTTT	60	215	ins44733
	R:TCTCTGGCATTGAAATGAGAAGC			
P11	F:CAGTGTGACCTCAATAGTCGGTAT	60	366	ins33514
	R:CTCTGCTAATGTCCCTGCCAT			
P12	F:ACTAAAAGAGACATCAGTGTGACC	59	464	ins33514
	R:TGCAGTCACACAGCTAGCAATA			
P13	F:TCAGCCTCCTTGAACTCTATGG	59	290	ins28794
	R:TCTTGCCTCAAAACTTTCAGACTT			
P14	F:ATTCAGCCTCCTTGAACTCTATGG	59	284	ins28794
	R:CAAAACTTTCAGACTTGATTAGGGC			
P15	F:GGGGAAACTGAGTCTTGTTCTG	59	327	ins6583
	R:ATCAACCCTGGGTATTCTTTGG			
P16	F:GCCCTGTTTATAAAGTGATGGCT	60	280	ins44733
	R:ATGAGAAGCCACACTCATTTGGT			
P17	F:TGCCCTGTTTATAAAGTGATGGCT	60	272	ins44733
	R:CACACTCATTTGGTTCTGTTCCA			
P18	F:CCTTTGTTTTATAGTTGTCTCGGTA	60	216	del41712
	R:TTTAGTAGTGGCATGTGAAGTCTAG			
P19	F:CCCTAATGCTTTCTGTAACTTTTAT	57	263	del41712
	R:TTTAGTAGTGGCATGTGAAGTCTAG			
P20	F:ACAGGAGACATTGGTCGCTG	60	144	qPCR of *ACSL5*
	R:CTGCGACACCAGACTACTCC			
P21	F:TGTTTGTGATGGGCGTGAACCA	60	142	qPCR of *GPADH*
	R:ATGGCGTGGACAGTGGTCATAA			

### 2.4 Bioinformatics analysis of the goat *ACSL5* gene

The sequences of DNA and amino acid were aligned using MEGA 5.1 (http://www.megasoftware.net/, last access: 20 May 2024) and BioXM 2.6 (Nanjing Agricultural University, Nanjing, China). The evolutionary tree was generated by MEGA 5.1 and the NCBI pairing comparison algorithm (http://www.ncbi.nlm.nih.gov/blast, last access: 20 May 2024).

### 2.5 Typing detection of the InDel locus of the goat *ACSL5* gene

The PCR amplification system for typing the InDel locus of the goat *ACSL5* gene was 12 µ L. This consisted of 6 µ L of 2 × EasyTaq^®^ PCR Super Mix (Transgene Biotechnology Co, Ltd.), 0.2 µ L primer (forward and reverse), 50 ng of DNAv sample, and RNase-free water, which were supplemented to 12 µ L. The PCR amplification procedure was as follows: pre-denaturation at 95 °C for 5 min, denaturation at 94 °C for 30 s, annealing for 30 s, extension at 72 °C for 45 cycles, and extension at 72 °C for 10 min. Agarose gel electrophoresis was conducted as follows: a 3.5 % agarose gel was prepared with the addition of a nucleic acid dye, and then 3 µ L of the PCR product was sampled and electrophoresed for 50 min at 120 V.

### 2.6 The expression pattern of the goat *ACSL5* gene

The primer P21 for mRNA expression analysis was designed based on the sequence of the goat *ACSL5* gene (NCBI: NC_030833.1) (Table 1). qPCR was conducted using a Roche LightCycler 96 Real-Time PCR system (Roche, Mannheim, Germany). The qPCR protocol was based on the methodology described in our previous study (Wu et al., 2023a).

### 2.7 Statistical analyses

The genotype and allele frequencies of the InDel locus in the goat *ACSL5* gene were calculated using Microsoft Excel. Genotype statistical analysis, polymorphism information content (PIC), and the Hardy–Weinberg equilibrium (HWE) test for the InDel locus were calculated using the MSR online software (http://www.msrcall.com/Gdicall.aspx, last access: 22 July 2024) (Klinkenberg et al., 2010). The relationship between goat genotypes and body measurement traits was analyzed using a general linear model. The observed data for each trait can be expressed as $Y_{i}=u+G_{i}+e$ , where $Y_{i}$ is the trait-measured data for each animal, u is the mean for each trait, $G_{i}$ is the effect of the genotype, and e is the random error.

The 2^−ΔΔ*C* *t*^ method was applied to calculate the expression levels of the *ACSL5* gene with *GAPDH* as the reference gene (Bi et al., 2021). Non-template control (NTC) was included, which contained ddH2O instead of the cDNA template. Analysis of variance (ANOVA) was employed to analyze the expression levels of the *ACSL5* gene between samples using SPSS 17.0.

## 3 Results

### 3.1 Genetic parameters associated with the 25 bp deletion of the InDel site in the *ACSL5* goat gene

**Figure 1:**
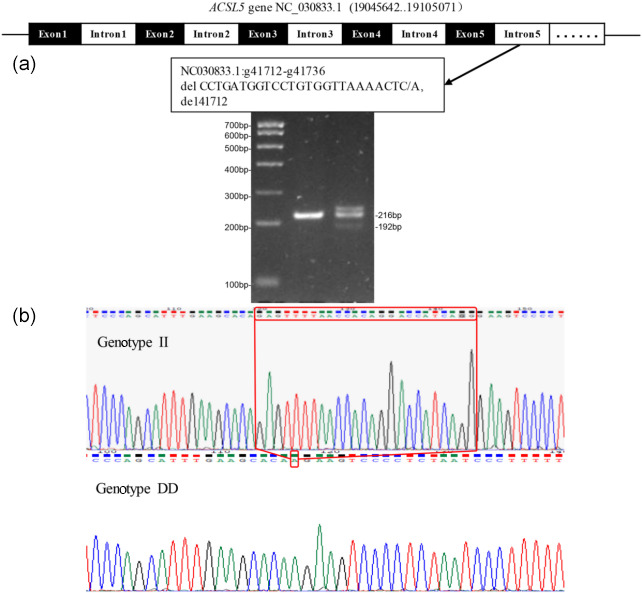
Electrophoretic profile **(a)** and sequencing peak map **(b)** of the goat *ACSL5* gene. Note: the electrophoretic profile uses marker I, genotype II = 216 bp, and ID = 216 and 192 bp. The red box of the sequencing peak map shows 25 bp insertion sequences.

Through the sequencing of amplified DNA pooling products, we identified prospective mutation sites in the *ACSL5* gene. Notably, a 25 bp insertion mutation site detected in the fifth intron of the gene, now designated as del41712 (NC_030833.1:g41712_41736 del CCTGATGGTCCTGTGGTTAAAACTC/A, del41712), was uncovered in the *ACSL5* gene. Two genotypes were identified in the test populations using direct 3.5 % agarose gel electrophoresis. There was one band (216 bp) for genotype II, and there were two bands (192 and 216 bp) for genotype ID (Fig. 1). The genotypes and allele frequencies, together with the genetic purity ( $H_{o}$ ), heterozygosis ( $H_{e}$ ), number of effective alleles ( $N_{e}$ ), and PIC at this locus, were also analyzed (Table 2). As displayed in Table 2, the genotypic frequencies in the three breeds ranged from 0.081 to 0.328 for genotype II and from 0.672 to 0.919 for genotype ID. The locus was moderately polymorphic (0.25 < PIC < 0.5) and was not in HWE in the test populations.

**Table 2:** Genetic polymorphism of InDel within the *ACSL5* gene.

Locus	Breed	Genotypic frequency		Allelic		$N_{e}$	$H_{e}$	PIC	HWE
	(size)	(individual quantity)		frequency					
		ID	II	D	I				
del41712	FQ ( n=123 )	0.919 (113)	0.081 (10)	0.459	0.541	1.9869	0.497	0.373	P<0.05
	NB ( n=286 )	0.825 (236)	0.175 (50)	0.413	0.587	1.9407	0.485	0.367	P<0.05
	JY ( n=119 )	0.672 (80)	0.328 (39)	0.336	0.664	1.8060	0.446	0.347	P<0.05

### 3.2 Construction of the phylogenetic tree

**Figure 2:**
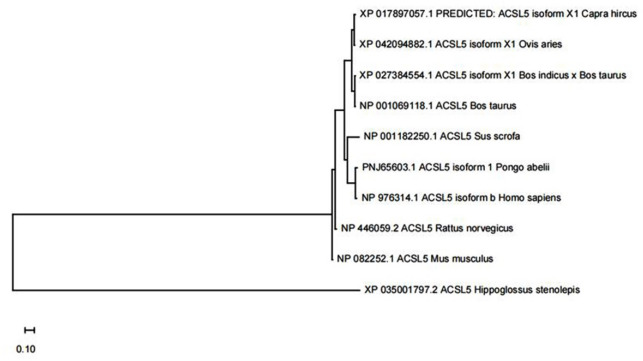
Phylogenetic analysis of the *ACSL5* gene in 10 species. Note: the phylogenetic tree was constructed from amino acid sequences using the MEGA 5.1 neighbor-joining method.

The goat *ACSL5* transcript encodes 683 amino acids. The results showed (Table 3) that the amino acid sequence of the goat *ACSL5* gene was similar to those of *Ovis aries* (XP 042094882.1), *Bos indicus* × *Bos taurus* (XP 027384554.1), *Bos taurus* (NP 001069118.1), *Sus scrofa* (NP 001182250.1), *Pongo abelii* (PNJ 65603.1), *Homo sapiens* (NP 976314.1), *Rattus norvegicus* (NP 446059.2), *Mus musculus* (NP 082252.1), and *Hippoglossus stenolepis* (XP 035001797.2), with similarities of 97 %, 92 %, 93 %, 80 %, 80 %, 80 %, 82 %, 81 %, and 64 %, respectively. The results of the phylogenetic tree showed that the amino acid sequence of the goat *ACSL5* gene was related closest to *Ovis aries* and was furthest from *Hippoglossus stenolepis* (Fig. 2).

**Table 3:** Accession numbers and descriptions of the amino acid sequences used in tree building.

Accession number	Description	Size (aa)
XP 017897057.1	ACSL5 *Capra hircus*	683
XP 042094882.1	ACSL5 isoform X1 *Ovis aries*	683
XP 027384554.1	ACSL5 isoform X1 *Bos indicus* × *Bos taurus*	683
NP 001069118.1	ACSL5 *Bos taurus*	683
NP 001182250.1	ACSL5 *Sus scrofa*	683
PNJ65603.1	ACSL5 isoform 1 *Pongo abelii*	683
NP 976314.1	ACSL5 isoform b *Homo sapien*s	683
NP 446059.2	ACSL5 *Rattus norvegicus*	683
NP 082252.1	ACSL5 *Mus musculus*	683
XP 035001797.2	ACSL5 *Hippoglossus stenolepis*	683

### 3.3 Expression profiles of the goat *ACSL5* gene

**Figure 3:**
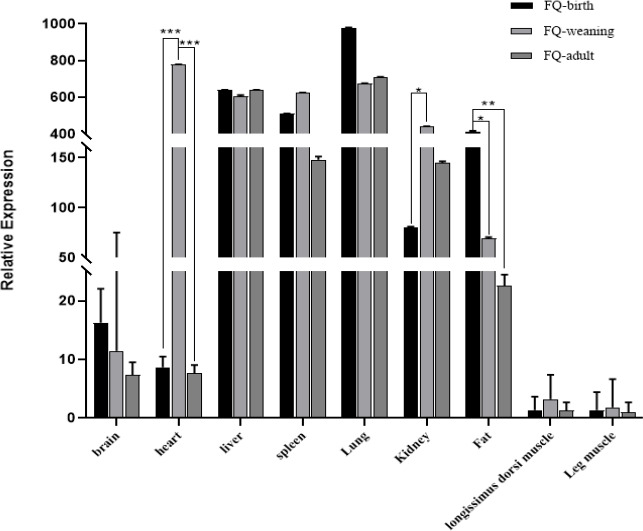
The mRNA expression patterns of the *ACSL5* gene. Note that the significance results refer to the three test methods: ^*^ representative ( P<0.05 ), ^**^ representative ( P<0.01 ), and ^***^ representative ( P<0.001 ).

The qPCR analysis indicated that the *ACSL5* gene was expressed ubiquitously in the nine tested tissues of FQ goats (Fig. 3). The results of mRNA expression in the leg muscles were used as a control. The expression level of the *ACSL5* gene is higher in liver, spleen, lung, kidney, and fat tissue, while its expression in the longissimus dorsi muscle was part of a lower trend. Although the means might decline, there is no statistical significance for the brain. Notably, when goats reach maturity, the expression levels of the *ACSL5* gene exhibit a progressive decline in the fat tissue. Variable expression patterns of the *ACSL5* gene were observed in all the collected tissues. In the heart tissue specifically, the expression of the *ACSL5* gene was significantly greater in the weaning period than in the birth and adult periods ( P<0.001 ). Additionally, in the fat tissue, *ACSL5* mRNA levels at birth were far higher than those observed in adulthood ( P<0.01 ) and were also greater than in the weaning period ( P<0.05 ). The expression levels in the kidney tissue differed substantially across the birth and weaning periods ( P<0.05 ).

### 3.4 Association analysis of different genotypes of the InDel locus of the *ACSL5* goat gene with body measurement traits

The analysis of the association between the InDel locus and the growth characteristics revealed a significant correlation with several body measurement traits in goats ( P<0.05 ) (Table 4). For the del41172 locus, individuals with II genotypes were larger than those with ID and ChD ( P=0.017 ) in the FQ breed and with BLI ( P=0.030 ) and ChCI ( P=0.007 ) in the NB breed. Individuals with ID genotypes were larger than those with II and BH ( P=0.012 ) in the NB breed.

**Table 4:** Relationship between the InDel locus of the *ACSL5* gene and body measurement traits in goats.

Breed	Body measurement trait	Mean ± SE		P value
		II	ID	
FQ	ChD	30.39 ± 0.31^a^	27.7 ± 1.51^b^	0.017
NB	BH	68.81 ± 0.37^b^	71.03 ± 0.72^a^	0.012
	BLI	95.20 ± 0.50^a^	92.64 ± 0.95^b^	0.030
	ChCI	126.41 ± 0.63^A^	122.44 ± 1.18^B^	0.007

Note: values with different superscripts within the same column differ significantly at P<0.05 (a, b) and P<0.01 (A, B). ChD: chest depth, BH: body height, BLI: body length index, and ChCI: chest circumference index.

## 4 Discussion

Transcriptome sequencing identified the candidate gene *ACSL5*, revealing a novel 25 bp deletion mutation in the fifth intron of the gene (NC_030833.1: g41712_41736 del CCTGATGGTCCTGTGGTTAAAACTC/A, del41712). In this study, the comparison of the similarities and the results of phylogenetic tree analysis indicate that the *ACSL5* goat gene has been preserved well throughout ruminant evolution. We performed genotyping on a cohort of 528 individual goats from three different breeds. The FQ goat is a local breed in Fujian Province, China, while the NB goat is a breed cultivated in Egypt. The JZ goat is a crossbreed developed from the Jianzhou native goat and the NB goat. The PIC results showed that the del41712 variant of the *ACSL5* gene had moderate polymorphism in all three goat breeds. Intriguingly, the DD genotype was absent in all three breeds. This absence leads us to speculate that the DD genotype may be less favorable compared to the other genotypes, which might explain its elimination through natural selection during goat evolution or purposeful breeding. Such evidence underscores the potential significance of the *ACSL5* gene for the growth and development of goats. These findings suggest that the genetic locus may be subject to substantial selection pressure and gene mutation within these breeds. Moreover, a significant departure from HWE was noted in these breeds, likely due to nonrandom mating resulting from population stratification, selection, or genetic drift (Janecka et al., 2021) as well as inbreeding, population substructures, or copy number variation (Chen et al., 2017).

The ACSL family comprises lipid metabolism enzymes that convert free fatty acids into bioavailable lipoyl coenzyme A. Within this family, the *ACSL5* gene plays a crucial role in the synthesis of triglycerides, phospholipids, and cholesterol as well as in the metabolism of fatty acids (Paul et al., 2014). This study is the first to reveal the expression patterns of the *ACSL5* gene in the nine tissues of FQ goats at three key developmental stages (birth, weaning, and adulthood) and explores the changes in gene expression in three important periods. Weaning is also a particularly important period in the growth and development of goats, because it can cause stress in goats that may have multiple effects on their physiological functions (Liao et al., 2021). In detail, the birth of kid goats is also a period that has a significant impact on their growth and development, as they move from body-circulation intake of nutrients to ingestion of nutrients. The *ACSL5* gene is expressed in all three stages in a goat's brain. The brain, a complex organ composed of several highly specialized and interacting structures, regulates various metabolic processes in the body, including food intake, energy expenditure, insulin secretion, liver glucose production, and glucose and fatty acid metabolism in fat tissue and skeletal muscle (Roh et al., 2016). Mutual regulation between the brain and other organs is essential for maintaining energy and glucose homeostasis. The *ACSL5* gene plays a crucial role in energy metabolism and fatty acid metabolism (Paul et al., 2014), and it is expressed in all three periods in the Fuqing goat brain, suggesting that the *ACSL5* gene may play an important role in brain regulation of fatty acid metabolism. Additionally, significant differences in fat expression were observed across the three periods ( P<0.05 ). Previous studies found that FQ goats have a high intermuscular fat content and that the *ACSL5* gene is actively involved in fatty acid metabolism, suggesting that the *ACSL5* gene may play an important role in fat deposition in FQ goats. In all three periods, the expression levels of the *ACSL5* gene in the liver tissues of FQ goats showed an increasing trend. The liver is the largest digestive gland and metabolic organ of mammals and plays an important role in the metabolism of amino acids, sugars, fats, and other substances as well as in protein and digestive fluid synthesis (Si-Tayeb et al., 2010). The liver also has a detoxification function (Kieffer et al., 2016). However, it is not fully developed at birth and gradually matures as it grows and develops (Reinke and Asher, 2016). The *ACSL5* gene is actively involved in lipid and carbohydrate metabolism, and we found that the *ACSL5* gene had higher expression levels in the three periods in Fuqing goat livers. This suggests that the *ACSL5* gene plays an important role in liver development and function. Notably, heart expression was significantly different at weaning compared to birth and adulthood ( P<0.001 ). However, the mechanism by which cardiac expression suddenly increases during weaning is not clear. These results suggest that the *ACSL5* gene plays a critical role in the energy metabolism, fatty acid deposition, and growth and development of goats.

Marker-assisted selection (MAS) is an indirect selection process that utilizes genetic markers within quantitative trait loci (QTL) to identify and select favorable traits, predict genotypes, and improve individual breeding performances. MAS has been widely adopted in animal breeding, particularly for traits that are challenging or expensive to measure and for recessive traits. MAS is the most suitable screening technique in these contexts (Wijayanti et al., 2022a). InDel is one of the many mutations that have been identified and is highly dense, accurate, and easy to genotype. Through association analysis between InDel sites and production traits, molecular markers with significant production traits are screened (Mills et al., 2006). Our previous studies reported that the InDel sites of key transcriptome candidate genes, e.g., the *CPT1a* gene (Li et al., 2021) and the *FGF7* gene in goats (Wu et al., 2023a), had significant effects on the body measurement traits of goats. In this study, the association analysis results between the del41712 locus of the *ACSL5* gene and body measurement traits showed a significant correlation with ChD in FQ goats as well as BH, BLI, and ChCI in NB goats. The findings indicate that BH and ChD directly influence the BW of goats, with both traits showing a positive association with BW (Qin et al., 2022). InDel in introns plays an important role in gene regulation and can produce genetic effects. Three InDel sites were identified in the intron of the *IGF2BP1* gene in goats (Liu et al., 2023), while two InDel sites were found in the intron of the *SNX29* gene in goats (Bi et al., 2022). These genetic variations have been shown to significantly impact their body measurement traits. Intron InDel can induce changes in transcription factor binding sites and may contain regulatory sequences for gene expression, transcriptional regulation and translation, and mRNA processing (Wijayanti et al., 2022b). The InDel site located in the third intron of the *AKAP12* gene in Shaanbei white cashmere goats has a significant impact on gene expression and is strongly associated with body weight, body length, chest depth, chest width, hip width, and chest circumference (Bai et al., 2021). An InDel site located in the first intron of the *BMPR1B* gene in Taihu pigs has been found to have regulatory effects on the expression of this gene in the endometrial tissue (Liu et al., 2022). Therefore, we speculated that the -25 bp InDel locus in the fifth intron affected the expression of the *ACSL5 *gene and the body measurement traits of goats. This study found that the del41712 locus of the *ACSL5* gene has a significant impact on multiple body measurement traits of goats. The previous studies demonstrated that the *ACSL5* gene plays a crucial role in the synthesis of triglycerides, phospholipids, and cholesterol as well as in the metabolism of fatty acids. Therefore, the del41712 site can be utilized as a genetic marker site that influences the body measurement traits of goats.

## 5 Conclusions

The *ACSL5* gene is highly expressed in the heart, liver, spleen, lungs, kidneys, and fat of FQ goats. As goats age, the expression levels of the *ACSL5* gene gradually decrease in the brain and fat tissues. The del41172 locus of the *ACSL5* gene in three breeds of goat was analyzed. The results of the association show that, of the three goat breeds examined, only two genotypes (II and ID) were identified in this InDel polymorphism. This genetic locus demonstrated a significant association with body measurement traits in both the FQ and NB goat breeds. The del41712 locus can be employed as a molecular marker locus affecting the body measurement traits of goats. This provides a research basis for breed improvement, with the objective of enhancing the body measurement traits of goats.

## Data Availability

The data presented in this study are available on request from the corresponding author.

## Appendix A Abbreviations

**Table:**

*ACSL5*	Acyl-CoA synthetase 5
*FGF7*	Fibroblast growth factor 7
*ITGAD*	Integrin subunit alpha D
ACSLs	Long-chain fatty acyl-CoA synthetases
MAS	Marker-assisted selection
RNA-Seq	Transcriptome sequencing technology
InDel	Insertion/deletion
II	Insertion/insertion
ID	Insertion/deletion
DD	Deletion/deletion
SPSS	Statistical Product and Service Solutions
bp	Base pair
INS	Intron
EX	Exon
PCR	Polymerase chain reaction
qPCR	Reverse-transcription quantitative real-time polymerase chain reaction
$C_{q}$	Quantification cycle
LSM ± SE	Least-square mean ± standard error
FQ	Fuqing goat
NB	Nubian goat
JZ	Jianzhou Daer goat
BW	Body weight
BH	Body height
BL	Body length
ChC	Chest circumference
ChD	Chest depth
ChW	Chest width
HuW	Hucklebone width
CaC	Cannon circumference
TI	Trunk index
BLI	Body length index
ChCI	Chest circumference index
CaCI	Cannon circumference index
CWI	Chest width index
HuWI	Hucklebone width index
